# Biomimetic nano-in-gel vaccine platform with spatiotemporal co-delivery capacity for DC maturation against immunosuppressive tumors

**DOI:** 10.1016/j.ijpx.2026.100662

**Published:** 2026-09-16

**Authors:** Jie Wu, Xinxin Bu, Zi Liu, Yupeng Zhou, Lingzhi Zhang, Zixuan Wang, Wenquan Cheng, Yanxia Yu, Huae Xu, Qun Gu

**Affiliations:** a Department of Pharmaceutics, National Vaccine Innovation Platform, School of Pharmacy, Nanjing Medical University, Nanjing 211166, People's Republic of China; b Department of Pharmacy, The Affiliated Suzhou Hospital of Nanjing Medical University, Suzhou Municipal Hospital, Suzhou, People's Republic of China; c Gusu School, Nanjing Medical University, Suzhou, People's Republic of China; d Department of Cardiovascular Surgery, People's Hospital of Kizilesu Kirgiz Autonomous Prefecture, Artux City, People's Republic of China; e Department of Cardiovascular Surgery, The First Affiliated Hospital with Nanjing Medical University, Nanjing 210029, People's Republic of China

**Keywords:** Cancer vaccine, Extracellular matrix hydrogel, Gastric cancer, PD-L1 siRNA

## Abstract

Therapeutic tumor vaccinations are promising for cancer immunotherapy. However, challenges like inadequate antigen encapsulation, weak immune activation, and an immunosuppressive tumor microenvironment limit their efficacy. To overcome these, an injectable Nano-in-Gel vaccine composed of porcine lung-derived extracellular matrix hydrogel (ECMG), tumor cell lysate (TCL), Poly I:C, and PD-L1 siRNA-loaded lipid nanoparticles (LNP) was developed. The ECMG derived from lung loose connective tissue exhibits excellent biocompatibility and degradability. It mimics the mechanical stress environment of natural ECM and contains bioactive components such as proteins, peptides, and polysaccharides. Administration of two weekly doses efficiently facilitated the transport of antigens and adjuvants to dendritic cells (DCs), thereby inducing robust immune responses and promoting dendritic cell maturation within lymph nodes. Importantly, PD-L1 siRNA-loaded LNPs reduced immunosuppressive signals in mature DCs, enhancing T-cell production and infiltration while alleviating the immunosuppressive tumor environment. The hydrogel scaffold achieves sustained antigen presentation and TLR3 activation through the sustained delivery of TCL and Poly I:C, while the targeted delivery characteristics of LNPs ensure the precise silencing of the immune checkpoint PD-L1 by siRNA. Overall, this study offers a proof-of-concept for a more efficient dendritic cell (DC)-activating strategy that integrates natural hydrogel scaffolds, whole tumor cell vaccines, and immune checkpoint inhibitors.

## Graphical abstract

**Figure:**
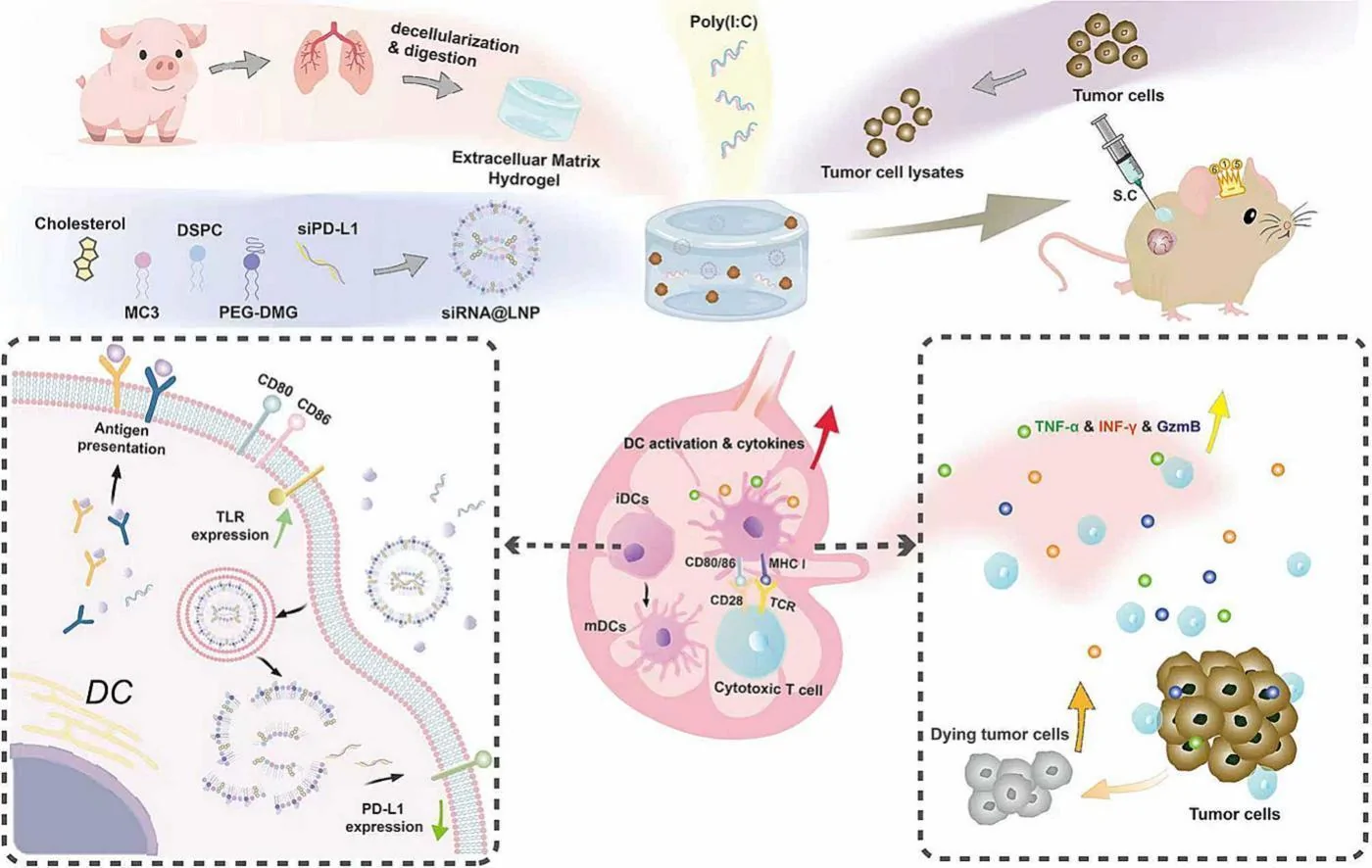

## 1 Introduction

Tumor cell lysate (TCL) vaccines, which provide a rich source of potential or known tumor antigens, represent a novel strategy for personalized cancer immunotherapy with great therapeutic potential(Adamik et al., 2023; Ding et al., 2021; Liu et al., 2022; Zhou et al., 2025). However, their *in vivo* immune efficacy still faces multiple challenges, including insufficient lymph node targeting, low antigen presentation efficiency, weak immunogenicity, and the immunosuppressive tumor microenvironment (TME)(Jou et al., 2021; McNeel, 2018; Morse et al., 2021). These issues collectively pose a significant obstacle to the effective activation of DCs, weakening the immune response of cytotoxic T lymphocytes (CTLs) and reducing the generation of memory T cells(Chen et al., 2022; Liu et al., 2024; Shen et al., 2024; Tian et al., 2020). Therefore, only tumor vaccines with a “delivery-activation-inhibition removal” trinity design concept can truly maximize their therapeutic effect, which encompasses multiple dimensions such as optimizing the antigen delivery system, enhancing the maturation and activation of DCs, and actively breaking through the immune suppression barrier(Christo et al., 2024; Corrado and Pearce, 2022; Huang et al., 2022; Ma et al., 2019).

Interestingly, recent studies have found that the extracellular matrix (ECM) is not only a scaffold for maintaining tissue morphology but also plays a significant role in regulating the microenvironmental signals. The various proteins contained in the extracellular matrix can be specifically cleaved by proteases to release small peptide fragments with specific biological activities, which are called “matrix peptides”. These small peptides can bind to various receptors on the cell surface, such as integrins, epidermal growth factor receptors, DDR receptors, *etc.*, conduct signal pathways, and regulate immune processes and cell migration, *etc.* Biomimetic materials based on naturally sourced matrix peptides are expected to be used as novel biomaterials in fields such as anti-tumor immunotherapy(Jariwala et al., 2022; Sivaraman and Shanthi, 2018). For instance, Ouyang et al. demonstrated that the VGVAPG oligopeptide (E motif) derived from ECM degradation has pro-inflammatory effects and can activate monocytes and macrophages through the component NEU1 of the elastin receptor complex(Yi et al., 2025). Additionally, Fotis Asimakopoulos and his colleagues revealed that matrix peptides derived from angiopoietin can regulate the differentiation of Batf3-dependent dendritic cells and promote T-cell infiltration in colorectal cancer(Papadas et al., 2020; Papadas et al., 2019). These studies suggest that matrix peptides are not only by-products of ECM degradation but also important signaling molecules that actively regulate immune responses, and are expected to become new functional modules in the design of therapeutic vaccines for tumors(Suckow and Hiles, 2023).

The spatiotemporal control of the ordered release of antigen and adjuvant components through nanoparticles can significantly enhance vaccine efficacy. For instance, engineered lipid nanoparticles (LNPs) can actively target and accumulate in the paracortical area of lymph nodes, improving antigen delivery efficiency; sustained-release systems such as hydrogels and nanocarriers can prolong antigen exposure time, reducing the risk of tumor recurrence. Spatiotemporal coupling carriers that combine targeted delivery and sustained antigen release are expected to further enhance immune activation(Liu et al., 2023). For example, Gao et al. designed a fully synthetic polymer-based hydrogel to improve vaccine efficiency(Gao et al., 2024), while Ji et al. developed a hydrogel based on methacrylated gelatin to achieve long-lasting immune activation(Ji et al., 2023). However, synthetic materials often fail to mimic the components of the natural ECM and its mechanical stress microenvironment, which may reduce DC activation and vaccine efficacy(Ho et al., 2022). In recent years, ECM-derived hydrogels (ECMG) have emerged as novel biomimetic thermosensitive materials with good biocompatibility and degradability(Spang and Christman, 2018). They can also simulate the mechanical stress environment of the natural extracellular matrix and contain various bioactive components such as proteins, peptides, and polysaccharides(Evangelista-Leite et al., 2023; Ma et al., 2024). Notably, lung tissue, rich in loose connective tissue and collagen components, is an ideal biological resource for preparing ECMG(Cherian et al., 2022).

As shown in Scheme 1, we have developed a novel cancer vaccine consisting of four components: ECMG, LNPs loaded with PD-L1 siRNA, TCL, and poly(I:C). These four components serve respectively as sustained-release carriers, immune checkpoint inhibitors, whole tumor antigens, and adjuvants. In the tumor-draining lymph nodes, the continuous release of TCL, poly(I:C), and LNP/siRNA can locally drive sustained DC activation and antigen cross-presentation, ultimately reversing the immunosuppressive tumor microenvironment and activating T cells.

**Scheme 1:**
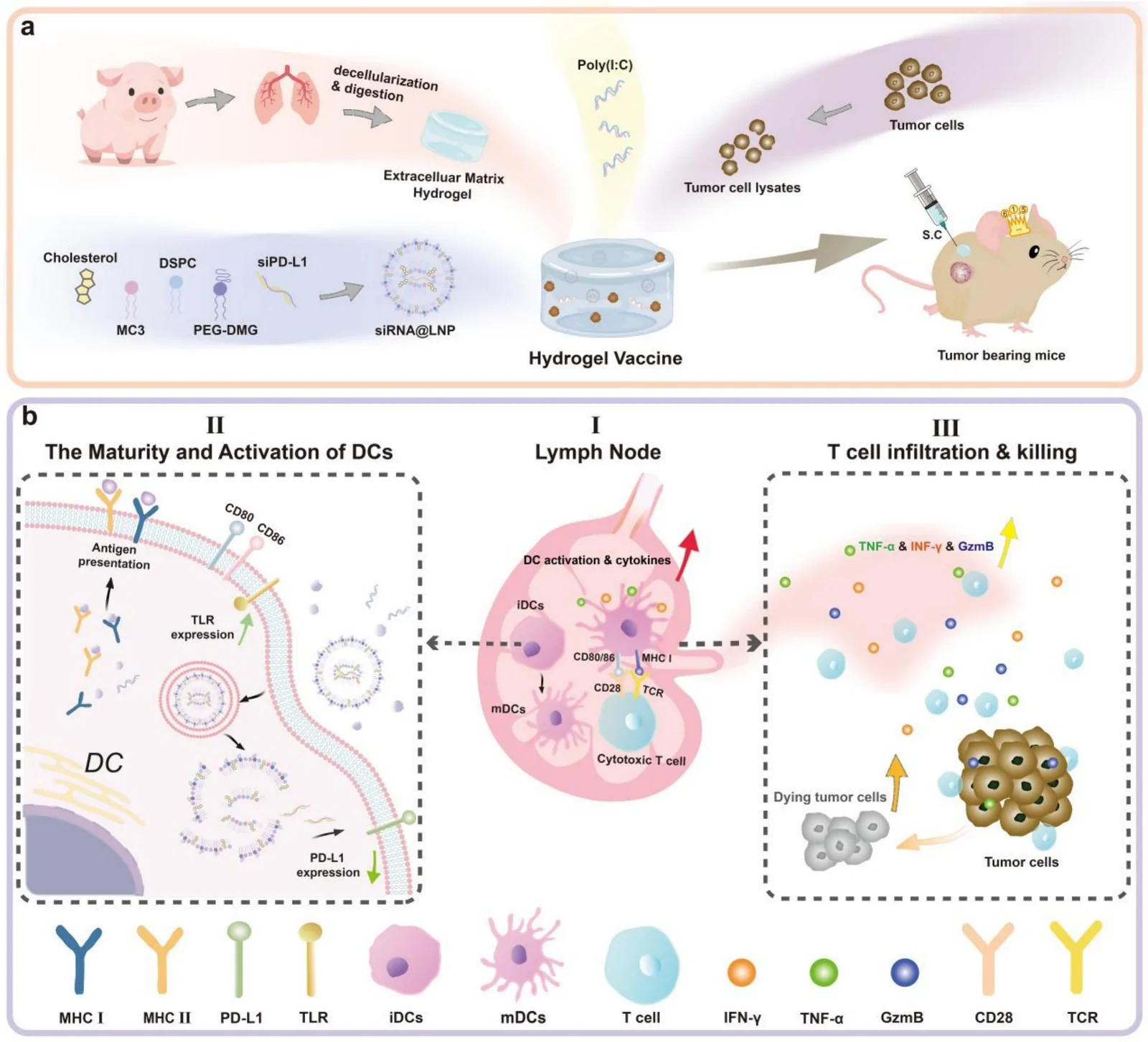
Schematic illustration of the preparation of ECM hydrogel vaccine and its mechanism for inducing robust antitumor immune responses. **(a)** The ECM hydrogel was derived from porcine lung tissue. LNP/siRNA nanoparticles were assembled with PD-L1 siRNA, MC3, cholesterol, DSPC, and PEG-DMG. TCL was prepared from MFC cancer cells. Mixing LNP/siRNA, TCL, poly(I:C), and pH-adjusted ECM solution forms a hydrogel at 37 °C. **(b)** Subcutaneous injection of the hydrogel vaccine induces DC activation and maturation, antigen-specific T-cell priming, and enhances anti-tumor immunity.

This new strategy has several key advantages. Firstly, the extracellular matrix hydrogel vaccine is injectable and has good biocompatibility, offering clinical application convenience, and can continuously stimulate DCs to enhance efficacy. Secondly, PD-L1 siRNA can effectively block PD-1 signaling, reverse immune suppression, and enhance cytotoxic T cell activity(Adamik and Butterfield, 2022; Giobbe et al., 2019; Liu et al., 2021; Wu et al., 2019; Xu et al., 2021; Yang et al., 2025). Thirdly, tumor cell lysate (TCL) has a wide antigen coverage and can maintain the natural conformation of antigens. More importantly, matrikines possess the natural extracellular matrix microenvironment and active components, which can further promote the maturation and survival of DCs and enhance antigen presentation in lymph nodes. Both *in vitro* and *in vivo* studies confirm the vaccine's robust antitumor effects. Overall, this study highlights the potential of spatial and temporal control of tumor vaccine kinetics and immune tolerance reversal through natural bionic hydrogel scaffolds for efficient tumor immunotherapy.

## 2 Materials and methods

### 2.1 Materials and animals

Porcine lung tissues were procured from local slaughterhouses. Pepsin was sourced from Shanghai Yeasen Biotechnology Co., Ltd., and siRNA was obtained from Hunan Aikerui Bioengineering Co., Ltd. All reagents essential for cell culture were supplied by Jiangsu KGI Biotechnology Co., Ltd. Compounds required for the induction of bone marrow differentiation in mice were acquired from Yeasen Biotechnology. Materials utilized in the preparation of lipid nanoparticles (LNP) were procured from AVT Pharmaceutical Technology Co., Ltd. Additionally, all flow cytometry antibodies were purchased from BioLegend. Deionized water from a Millipore purification apparatus (resistivity >18.2 MΩ cm) was used in all experiments. All experimental animals were housed in a pathogen-free environment, and had unrestricted access to food and water throughout the study. The Animal Care and Use Committee of Nanjing Medical University approved all animal experiments (IACUC-2507020).

### 2.2 Preparation and characterization of extracellular matrix hydrogel

Healthy porcine lungs were obtained from adult pigs (weighing 80–100 kg) at a local slaughterhouse. Decellularization was carried out by adapting a previously established protocol. Briefly, lungs were sliced into 1 mm-thick sections, washed three times with deionized water to remove blood, and subjected to 0.5% SDS treatment with continuous agitation for 48 h. Then the SDS solution was replaced every 12 h. Slices were thoroughly washed with deionized water to eliminate any remaining SDS, subsequently frozen at −80 °C, and then freeze-dried to produce the decellularized lung ECM. Histochemical analysis using HE staining confirmed the extent of decellularization. The ECM material was enzymatically digested with pepsin that was dissolved in 0.1 M hydrochloric acid (HCl) at a concentration of 1 mg/mL, after which the ECM concentration was adjusted to reach a final concentration of 15 mg/mL. Digestion was carried out at room temperature with slow stirring for 48 to 60 h. The homogeneous liquid obtained was adjusted to pH 7.4 with sodium hydroxide and then diluted to the desired concentration with 10-fold phosphate buffer. The solution was stored at 4 °C. To optimize the experimental protocol, the effects of parameters such as gelation temperature, gelation time, salt concentration, or pH value on rheological properties were investigated. The swelling, erosion, *in vitro* degradation, and *in vivo* degradation properties of the extracellular matrix hydrogel were also examined.

### 2.3 Rheological property evaluation

The rheological properties of the extracellular matrix hydrogel, including the storage modulus (G′) and loss modulus (G′′), were investigated using a rheometer (MARS60, Thermo Fisher Scientific, Germany)(Falcone et al., 2023). In the strain sweep test, the frequency was fixed at 1 Hz and the strain range was from 1% to 100%. For the dynamic frequency sweep, the strain was fixed at 1% and the test was conducted within the frequency range of 1 to 100 Hz.

### 2.4 Swelling test

The swelling properties of the extracellular matrix hydrogel were characterized by the gravimetric method, referring to the literature(Lei et al., 2022). The freeze-dried hydrogel samples were taken as the initial mass (W₀), and then immersed in ultrapure water at 37 °C and 25 °C respectively. At the predetermined time points, they were taken out, the excess water was absorbed with filter paper, and then re-weighed as Wₜ. The equilibrium swelling ratio was calculated as:

$$\text{Swelling ratio}\;\left(\%\right)=\frac{W_{t}-W₀}{W₀}\times 100\%$$

### 2.5 Erosion test

Erosion properties were determined by immersing hydrogels in PBS (pH 7.4, 6.5, and 5.5). Fresh hydrogels were weighed as W₀, incubated in 1 mL PBS at 37 °C with shaking (60 rpm), and weighed periodically as Wₜ until complete dissolution(Bo and Wang, 2024). Erosion ratio was calculated as:

$$\text{Erosion ratio}\;\left(\%\right)=\frac{W₀-\mathrm{Wₜ}}{W₀}\times 100\%$$

### 2.6 *In vitro* degradation study

Enzymatic degradation was evaluated by incubating hydrogels in collagenase type I solutions (0.5, 1, and 2 mg/mL) at 37 °C with shaking (60 rpm). Samples were removed, dried, and weighed as Wₜ at desired timepoints. Degradation percent was calculated as:

$\text{Degradation percent}\;\left(\%\right)=\frac{W₀-\mathrm{Wₜ}}{W_{0}}\;$× 100%.

### 2.7 *In vitro* BMDC isolation and activation/maturation

Bone Marrow-Derived DCs were obtained from the hind limb bones of male C57BL/6 mice according to established protocols(Battais et al., 2024; Leblanc-Hotte et al., 2023). In brief, the distal ends of both bones were removed, and the marrow cells were extracted by flushing with RPMI 1640 medium. Erythrocytes were lysed, and the residual cells were collected by centrifugation at 1500 rpm for 10 min. The cells were resuspended to a concentration of 2 × 10^6^ cells per milliliter in RPMI 1640 medium containing 10% fetal bovine serum, 1% penicillin-streptomycin, 20 ng/mL of GM-CSF, and 10 ng/mL of IL-4, and then incubated in 12-well culture plates. Half of the culture medium was refreshed with new RPMI-1640 and additional cytokines were included during the incubation period. The cells were ready for collection by day 5 to 7.

### 2.8 Biological safety test

Cytotoxicity of ECM hydrogel was assessed using Zombie-labeled DCs analyzed by flow cytometry (FACSymphony A5 SORP, BD, USA). Isolated DCs were maintained in RPMI-1640 medium enriched with 10% fetal bovine serum and 1% penicillin-streptomycin at 10^4^ cells/mL in 24-well plates containing varying concentrations of ECM hydrogel (0, 1, 5, 10, 20 mg/mL). After co-incubation for 24, 48, and 72 h, respectively, DCs were collected for analysis. *In vivo* biosafety was evaluated by subcutaneously injecting blank hydrogels (100, 200, and 300 μL) into male C57BL/6 J mice. Subcutaneous nodules were measured over time, and tissues were isolated upon complete degradation, fixed in 4% paraformaldehyde, and subsequently stained using HE. Hepatorenal function was monitored *via* serum levels of AST, ALT, T-BIL, BUN, and CREA, showing no significant changes 6 days post-treatment. HE staining of major organs (heart, liver, spleen, lung, kidney) was conducted to assess tissue damage.

### 2.9 Preparation and characterization of LNP/siRNA

LNP/siPD-L1 was prepared using the solvent injection method. An organic solution was formulated in ethanol, comprising the ionizable lipid DLin-MC3-DMA (MC3), DSPC, cholesterol, and PEG2000-DMG at a molar ratio of 50: 10: 38.5: 1.5. The aqueous solution consisted of siRNA in citrate buffer (pH 4.0). Mixing the phases at various N/P ratios (nitrogen in MC3 to phosphate in siRNA) using the ethanol dilution method formed LNP/siRNA. Particle size and zeta potential were determined employing a nanoparticle analysis system (BeNano 90 Zeta, Bettersize, China), and morphology was observed *via* TEM (JEM-1400Flash, JEOL, Japan).

### 2.10 Preparation of tumor cell lysates (TCL)

Murine gastric carcinoma cells (MFC) were cultured in serum-containing RPMI-1640 medium(Wang et al., 2019). Cells were enzymatically dissociated using 0.25% trypsin, rinsed with chilled PBS, and resuspended at 1 × 10^7^ cells/mL. Aliquots underwent five cycles of freezing and thawing (liquid nitrogen snap-freezing followed by 5-min thawing at 37 °C in a water bath). Clarified lysates were obtained by cold centrifugation (4 °C), and protein content was quantified using the Coomassie Brilliant Blue (CBB) assay.

### 2.11 Construction and evaluation of hydrogel-derived cancer vaccine preparations

Typically, the ECM solution was adjusted to a neutral pH by adding 1 M NaOH at a digestion-to-neutralization volume ratio of 1:10, followed by the addition of 1/9 of the final volume of 10× phosphate-buffered saline (PBS). TCL (100 μg), poly(I:C) (100 μg), and LNP/siRNA (8 μg siRNA) were added to 1 mL of adjusted ECM solution and stirred for 5 min to form a homogeneous formulation. For the characterization of physicochemical properties, rheological measurements were conducted using a MARS60 rheometer to evaluate mechanical property changes before and after cargo encapsulation. For LNP/siRNA release characterization, FAM-labeled RNA liposome nanoparticles were incorporated into hydrogel, which was immersed in collagenase type I solution. Supernatant was collected at predetermined intervals, replaced with fresh solution, and fluorescent signal was detected with a fluorescence plate reader.

### 2.12 Modulation of bone marrow-derived DCs *in vitro*

Fully differentiated BMDCs were incubated with either free TCL, poly(I:C), LNP/siRNA, or hydrogel-encapsulated delivery systems. By day seven, suspension cells displayed typical dendritic features and underwent staining procedures using anti-mouse MHC-I (H-2Kb)-PE, anti-mouse MHC-II-PE-Cy7, anti-mouse CD11c-BV785, anti-mouse CD86-AF700, anti-mouse CD80-BV711, anti-mouse PD-L1-APC, and anti-mouse Live/Dead-APC-Cy7. Antibodies were used at a concentration of 1 μg per 2 × 10^6^ cells, and staining was performed for 30 min. Cells were fixed with 4% PFA, resuspended in 1× PBS, and examined using flow cytometry (FACSymphony A5 SORP, BD, USA). The percentage of cells indicating the presence of each protein was recorded, with CD11c serving as the foundation for enumerating double-positive DCs.

### 2.13 *In vivo* immunization assay

Six-week-old male 615 mice were immunized *via* subcutaneous injection on days 0 and 7 with various formulations. These formulations included free TCL (100 μg per mouse), poly(I:C) (100 μg per mouse), siRNA (10 μg per mouse), or combinations of these components delivered in a hydrogel (200 μL). Lymph nodes were harvested from both immunized and control mice on the seventh day after the last immunization. Then the lymph nodes were gently ground between two sterile glass slides to release cells. To investigate the activation of mature DCs in lymph nodes, cells isolated from these tissues were labeled by incubating with fluorescently labeled primary antibodies. Specifically, staining with BV785-conjugated CD11c antibodies was performed to assess dendritic cell infiltration and maturation, along with AF-700-conjugated CD86 antibodies, BV711-conjugated CD80, PE-conjugated MHC-I, and PE-Cy7-conjugated MHC-II antibody. Additionally, APC-conjugated PD-L1 antibody labeling was carried out to measure PD-L1 expression in DCs and BUV395-conjugated CD45, FITC-conjugated CD3, and BUV737-conjugated CD8 antibody labeling was conducted to assess T cell infiltration. Cells were selected based on their expression of CD11c, MHC-I, MHC-II, CD80, CD86, CD45, CD3, CD8 staining. The analysis was restricted to live dendritic cell events, and gating quadrants were established using samples stained with isotype control antibodies.

### 2.14 Antitumor efficacy of vaccine formulations based on hydrogels

615 mice (male, 6 weeks) were purchased from the Vital River Laboratory. The mice received subcutaneous injections of MFC cells (5 × 10^5^ cells per mouse in 1 × PBS) in the lateral aspect of the right leg to establish tumor models 8 days before treatment(Shen et al., 2022). Mice were assigned to nine experimental groups, and each received two doses of treatment on days 8 and 14 (*n* = 5 per group) *via* subcutaneous injection adjacent to the lymph nodes using a syringe fitted with a 25G needle. The groups were as follows: PBS (saline), free LNP/siRNA (10 μg PD-L1 siRNA per mouse), free LNP/NC-siRNA (10 μg negative siRNA per mouse), free TCL with poly(I:C) (TP, 100 μg per compound per mouse), free TCL with poly(I:C) and LNP/siRNA (TPL, 100 μg TCL, 100 μg poly(I:C), and 10 μg PD-L1 siRNA per mouse, respectively), blank ECM hydrogel (Gel, 200 μL per mouse), LNP/siRNA in hydrogel (Gel + LNP, 10 μg PD-L1 siRNA per mouse in 200 μL hydrogel), TCL with poly(I:C) in hydrogel (Gel + TP, 100 μg per compound per mouse in 200 μL hydrogel), or TPL in hydrogel (Gel + TPL, 100 μg TCL, 100 μg poly(I:C), and 10 μg PD-L1 siRNA per mouse, respectively, in 200 μL hydrogel). A second injection was administered 6 days later. Each group consisted of five mice. Mice were monitored every two days for signs of pain or distress, tumor growth, and changes in body weight. Tumor volumes (V, mm^3^) were calculated as V = length × width^2^ / 2 (de Figueiredo et al., 2023). Conventional hematological indicators were detected by enzyme-linked immunosorbent assay (ELISA). At the end of the experiment, tumor blocks were taken, homogenized, and filtered through a 70-μm cell sieve. Tumor-infiltrating lymphocytes (TILs) were washed with PBS, placed on 4 mL of Ficoll, and then covered with 1 mL of RPMI 1640. After centrifugation at 800 *g* for 30 min, the components at the corresponding density gradient were collected. TILs were harvested from the interface, washed, and stained with anti-mouse CD3-APC-Cy7, anti-mouse CD45-BUV396, anti-mouse CD8-BUV737, anti-mouse CD44-BV605, anti-mouse IFN-γ-BV711, anti-mouse CD62L-PE-Cy7, and anti-mouse GzmB+-BV421 antibodies, followed by flow cytometric analysis.

### 2.15 Statistical analysis

Statistical analyses were performed using GraphPad Prism 9.5.1. Results are presented as mean ± SEM from at least three independent experiments. For antitumor studies, each group included five mice. *p*-values were calculated using one-way ANOVA with Tukey post-tests as indicated. Statistical significance was defined as *p* < 0.05 (ns: *p* > 0.05, *p < 0.05, **p < 0.01, ***p < 0.001, ****p < 0.0001). Figures were designed using Adobe Illustrator.

## 3 Results

### 3.1 Preparation and characterization of thermosensitive ECM hydrogel

The ECM was successfully extracted from porcine lungs by the SDS chemical decellularization method using a reported method with slight modifications(Singelyn et al., 2009; Song et al., 2023). The obtained freeze-dried ECM powder was light red (Fig. 1a,b). To quantitatively evaluate the decellularization effect, HE staining was performed to compare the native and decellularized lung tissue samples. The results showed that no nuclear components were observed in the decellularized porcine lungs, confirming that the cells had been successfully removed (Fig. 1c,d). To explore the conditions for hydrogel formation, the effects of different ECM concentrations, ionic strengths, pH values, and temperatures on gelation were investigated (**Fig. S1, Supporting Information**). The optimal conditions were as follows: pH 7.4, 37 °C, an ECM digestion solution concentration of 15 mg/mL, which could form a hydrogel within 4 to 5 min. The hydrogel formed under these conditions was used for subsequent experiments (Fig. 1e). Interestingly, ECM hydrogel could not be formed at 45 °C, which might be due to protein denaturation caused by the high temperature (**Fig. S2, Supporting Information**). The storage modulus (G′) in rheology reflects the elastic (solid-like) properties of the gel, while the loss modulus (G″) indicates its viscous (liquid-like) characteristics. The rheometer test results showed (Fig. 1f) that within the strain range of 1% to 100%, G′ and G″ did not change significantly, indicating a linear viscoelastic behavior. Additionally, the viscosity remained stable within the range of 1% to 10% (Fig. 1g). Within the frequency range of 0.1 to 100 Hz, G was significantly higher than G″, suggesting that an elastic-dominated hydrogel network was formed (Fig. 1h**)**.

**Fig. 1:**
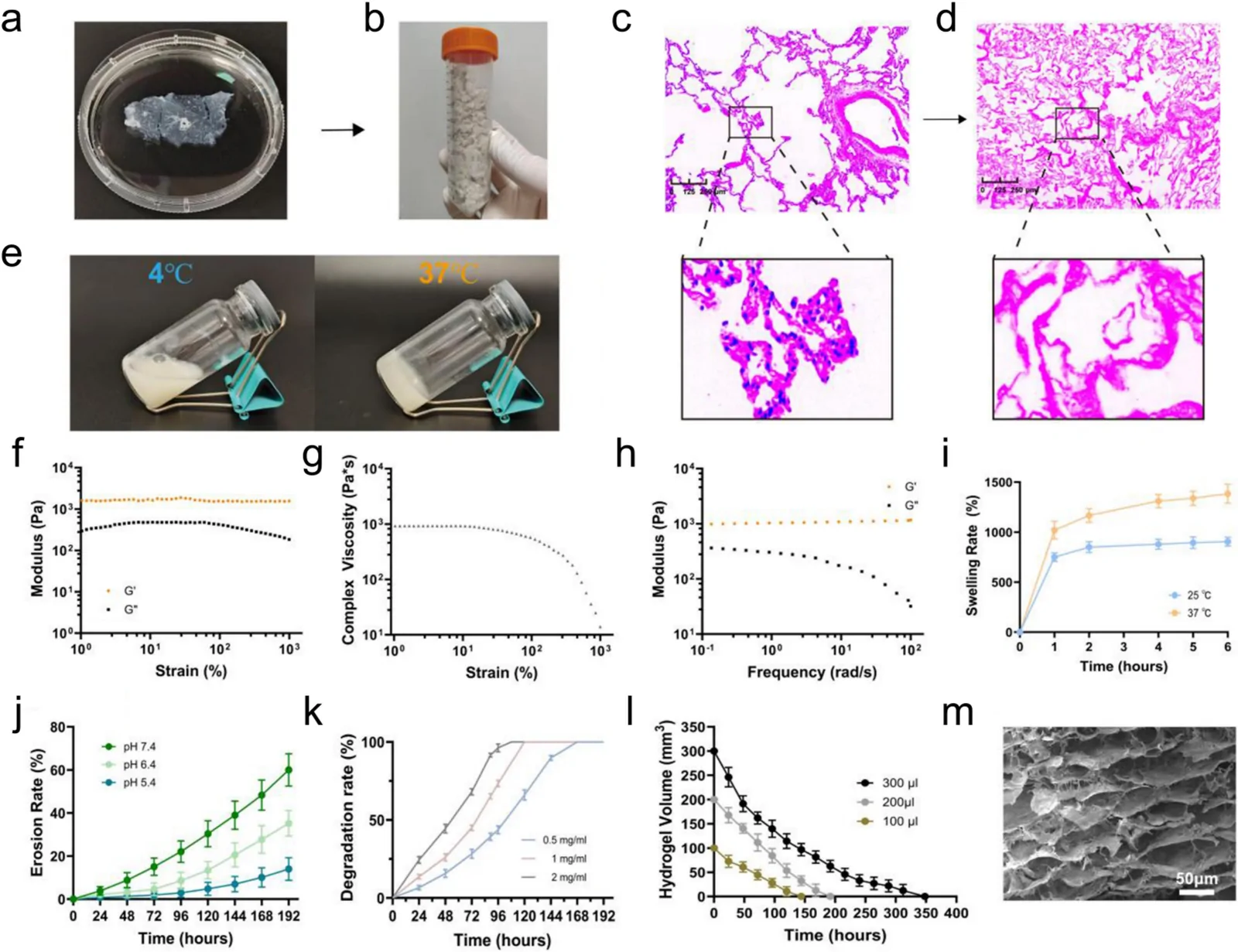
Fabrication and analysis of ECM hydrogel. **(a-b)** Illustrating the preparation process of ECM hydrogel. **(c-d)** Representative hematoxylin and eosin (HE) staining images of the lung tissue samples before (c) and after (d) decellularization. Scale bar = 125 μm. **(e)** The ECM solution forms a hydrogel upon heating at 37 °C. **(f)** Rheological analysis of ECM hydrogel under strain sweep. **(g-h)** Viscousness and Rheological analysis under dynamic frequency sweep. **(i)** Swelling rate of ECM hydrogel at 25 °C and 37 °C (*n* = 3). **(j)** Accelerated erosion test of ECM hydrogel at 37 °C using a shaker (*n* = 3). **(k)***In vitro* degradation of ECM hydrogel with varying collagenase I concentrations (*n* = 3). **(l)***In vivo* degradation of ECM hydrogel with different injection volumes. **(m)** Cross-sectional SEM image of ECM hydrogel.

The hydrogel swelling experiments demonstrated that the ECM hydrogel swelled significantly faster at 37 °C compared to 25 °C, with a swelling ratio reaching approximately 1300% (Fig. 1i). Interestingly, the ECM hydrogel exhibited different erosion patterns under different pH conditions. Under static conditions, ECM hydrogel degraded slowly, taking about 70 days to completely degrade, indicating that the hydrogel has certain stability without enzymatic degradation. Additionally, we conducted shaking acceleration experiments to test the degradation performance of the hydrogel in PBS at different pH values. After 8 days in PBS, the erosion rates at pH 7.4, pH 6.4, and pH 5.4 were 60%, 36%, and 17%, respectively, suggesting that pH affects the erosion rate (Fig. 1j). Under physiological pH conditions (7.4), the degradation kinetics of the hydrogel was the fastest. We speculate that this might be due to the enhanced permeability of PBS at this specific pH value, allowing it to more easily penetrate the hydrogel's structural matrix and mediate hydrolysis, thereby accelerating erosion.

The moderate degradation rate of hydrogels *in vivo* is of great significance for the sustained release of drugs and their biocompatibility(Lei et al., 2025). As shown in Fig. 1k, under *in vitro* simulated conditions, the ECM hydrogel exhibited good degradability under the action of different concentrations of collagenase (an enzyme naturally present in the human body). When exposed to a collagenase concentration of 0.5 mg/mL, nearly 90% of the ECM hydrogel had degraded after 144 h. We injected the hydrogel subcutaneously into mice to evaluate the *in vivo* degradation kinetics and determine the optimal injection volume and treatment interval. As shown in Fig. 1l, when 100 μL of hydrogel was injected subcutaneously, it was almost completely degraded within 4 days. When the injection volume was increased to 200 μL, it was completely degraded within 6 days. Interestingly, when the dosage reached 300 μL, the degradation rate of the hydrogel was relatively fast in the first two days and then gradually slowed down, taking approximately 13 days to be completely degraded. We speculate that the longer degradation period of the 300 μL hydrogel might be due to the rapid secretion of collagenase triggered by the stimulus and the subsequent depletion of the enzyme, ultimately reducing the degradation rate. To achieve the best sustained stimulation effect of the tumor vaccine, an injection volume of 200 μL is considered the optimal choice. Additionally, the internal structure of the hydrogel was further investigated by SEM analysis (Fig. 1m). The analysis indicated that the ECM had a moderate porosity with pore diameters of approximately 50 μm and a more uniform pore structure distribution. This porous structure offers greater advantages in regulating drug release(Gomari et al., 2025).

### 3.2 Preparation and characterization of ECM hydrogel vaccine

Based on our previous work(Li et al., 2024), we successfully constructed LNP/siPD-L1. The organic phase included DLin-MC3-DMA, DSPC, cholesterol, and PEG2000-DMG blended at a molar ratio of 50:10:38.5:1.5. The aqueous phase was prepared in a citrate buffer (pH 4.0) for nanoparticle self-assembly. LNP/siRNA complexes were formed by ethanol dilution-mediated mixing of the organic and aqueous phases at an N/P ratio of 5. The nanostructure of LNP/siRNA was observed by TEM (Fig. 2a), presenting a spherical form. LNP/siPD-L1 had an average particle size of 120 ± 0.35 nm (Fig. 2b) and a zeta potential of 4.7 ± 0.46 mV (Fig. 2c). Stability studies in isotonic PBS showed no significant particle size changes over 7 days (Fig. 2d). Whole-cell lysates were prepared *via* five freeze-thaw cycles, and protein concentrations were quantified using the Coomassie Brilliant Blue (CBB) assay with a BSA standard curve. The ECM hydrogel-based vaccine was fabricated by blending components. To maintain gelation, the ECM solution concentration was increased to 20 mg/mL before adding TCL (100 μg), poly(I:C) (100 μg), and LNP/siRNA (8 μg). These were sequentially introduced into pH-adjusted ECM solution under sterile conditions, followed by homogenization on ice.

**Fig. 2:**
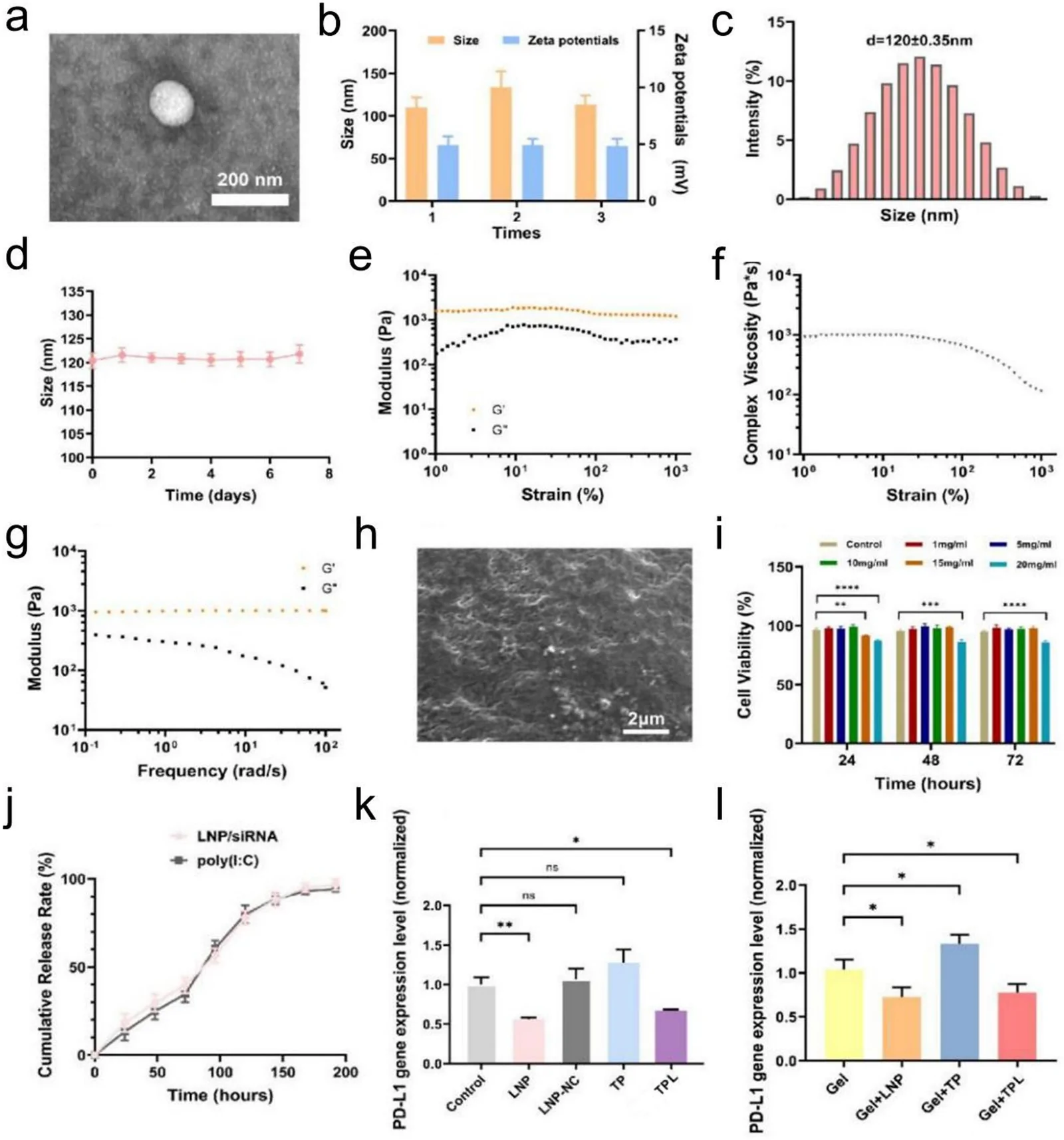
The characterization of LNP/siRNA and hydrogel vaccine. **(a)** TEM image of LNP/siRNA at N/P ratio 5. Scale bar: 200 nm. **(b)** Particle sizes and zeta potentials of LNP/siRNA from different production batches. **(c)** Average particle size: d = 120 ± 0.35 nm. **(d)** Size stability of LNP/siRNA in 1 × PBS over time (*n* = 3). **(e)** Rheological characterization of the hydrogel vaccine under strain sweep testing. **(f)** Viscosity characteristics of the ECM hydrogel vaccine. **(g)** Rheological analysis in dynamic frequency sweep experiments. **(h)** Cross-sectional SEM images of ECM hydrogel vaccine. **(i)** Viability of DCs subjected to different ECM hydrogel concentration treatments (*n* = 5). **(j)** Cumulative release rates of TCL, poly(I:C), and LNP/siRNA (*n* = 3). **(k)** Effects of the naked drug groups on mRNA-level gene silencing (*n* = 5). **(l)** Effects of hydrogel-treated groups on mRNA-level gene silencing (*n* = 5). Data are presented as mean ± s.d. Statistical significance was determined using one-way ANOVA with Tukey's test. *p < 0.05, **p < 0.01, ***p < 0.001, ****p < 0.0001; ns: no significant difference.

Physicochemical characterization confirmed structural integrity and functional competency as a therapeutic platform. Rheological analysis confirmed hydrogel integrity post-encapsulation. Under oscillatory strain (1–1000%), G' exceeded G", indicating retained structure (Fig. 2e). Within the strain range of 1% to 10%, the hydrogel is in a linear viscoelastic state, with its viscosity remaining stable. However, at higher strains, it transitions to shear-thinning behavior (Fig. 2f). Frequency-dependent analysis (0.1–100 rad/s) showed G' dominance, confirming a fully crosslinked elastic network (Fig. 2g). SEM evaluation revealed preserved dECM fibrillar architecture (Fig. 2h). Cytocompatibility tests of ECM hydrogel showed no toxicity to MFC and DC cells at ECM concentrations of 1–15 mg/mL (Fig. 2i) after 24 h.

Cumulative release profiles of ECM Hydrogel demonstrated sustained release exceeding 80% within 5 days (Fig. 2j). qPCR analysis of DC cultured with different formulations confirmed silencing ability. Compared with the negative control siRNA (LNP-NC) which did not reduce the expression of PD-L1 mRNA, LNP-siRNA significantly decreased the expression of this mRNA (*p* < 0.01), confirming the sequence-specific activity. In contrast, TP significantly increased PD-L1 mRNA expression (*p* < 0.05), likely attributable to TCL stimulation. However, vaccines that contain both TP and LNP-siRNA could decrease the expression of PD-L1, because of the function of siRNA (Fig. 2k). When encapsulated by hydrogel, LNP and Vaccine showed consistent results, indicating the inhibition effect of the hydrogel vaccine on PD-L1 (Fig. 2l).

### 3.3 *In vitro* ECM hydrogel-based vaccine activates and matures DCs

During activation and maturation, DCs upregulate markers such as CD11c, CD80, CD86, MHC-I, MHC-II, and PD-L1 expression(Lu et al., 2022). These activated DCs take up and internalize antigens, and present the resulting peptides to T cells: CD8+ T cells *via* MHC-I and CD4+ T cells *via* MHC-II(Ni et al., 2020). We explored whether the release of cargo from the hydrogel-formulated vaccine has the potential to stimulate and induce maturation of DCs (Fig. 3a). Cells were then seeded into the hydrogel-containing wells for co-culture under predetermined conditions (Fig. 3b). To assess DC phenotype and function, we measured the expression of these activation markers by flow cytometry following treatment with various formulations (including naked drugs and hydrogel-based groups).

**Fig. 3:**
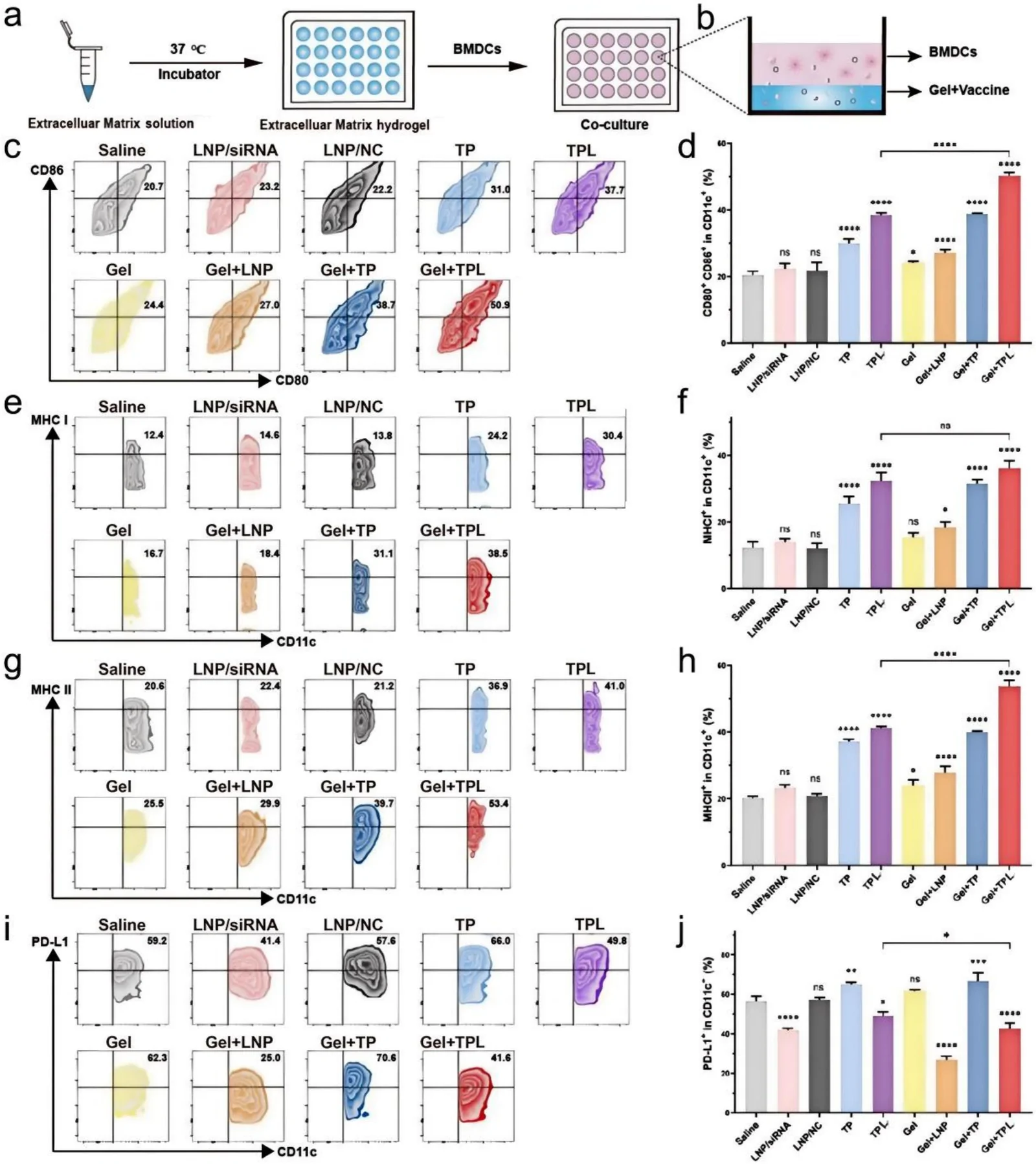
The immune activation of DCs *in vitro*. **(a)** Schematic illustration of the procedure of the cocultivation. **(b)** Schematic illustration of DCs co-incubated with hydrogel vaccine groups. **(c)** Flow cytometry diagrams and **(d)** corresponding quantitative assessment of the expression levels of CD80 and CD86 on DCs (*n* = 3). Flow cytometry diagrams and corresponding quantitative assessment of the expression levels of **(e-f)** MHC-I, **(g-h)** MHC-II, and **(i-j)** PD-L1 on DCs (*n* = 3). Data are presented as the mean ± s.d. Statistical significance was calculated *via* one-way ANOVA with Tukey's test. *p < 0.05, **p < 0.01, ***p < 0.001, ****p < 0.0001; ns: no significant difference.

As shown in Fig. 3c, the combination of TP and Vaccine significantly enhanced DC vitality and maturation compared to Saline. Hydrogel-based formulations (Gel+LNP/siRNA, Gel+TP, and Gel+TPL) achieved higher marker expression levels, outperforming free drug groups. Compared to Saline, Gel+TPL increased CD80 and CD86 expression by 30.2%, while Gel, Gel+LNP/siRNA, and Gel+TP groups increased expression by 3.7%, 7%, and 18.0%, respectively (Fig. 3d). ECM hydrogel likely enhances DC maturation, improving vaccine immunogenicity. MHC-I and MHC-II levels were upregulated in DCs treated with TP, Vaccine, and hydrogel groups (Fig. 3e-h). Compared to the vaccine, Gel+TPL increased MHC-I and MHC-II expression by 8.5% and 12.4%, respectively. Gel+TPL demonstrated 3.1- and 2.6-fold higher MHC-I and MHC-II expression than Saline. Blank hydrogel also showed significant MHC-I upregulation compared to saline (*p* < 0.05).

After antigen uptake, DCs may upregulate the expression of PD-L1, which serves as a feedback mechanism for immune regulation and can limit the cytotoxicity of T cells(Sprooten et al., 2024). Antigen internalization triggers DC maturation *via* pathogen-associated molecular patterns (PAMPs) or cytokine signals (*e.g.*, IFN-γ), which induce PD-L1 transcription through pathways like NF-κB or JAK/STAT(Oba et al., 2021). As shown in Fig. 3i, PD-L1 siRNA blocked the immune checkpoint, reducing PD-L1 expression by 35% in Gel+LNP compared to Saline (Fig. 3j).

Altogether, these results confirm sustained LNP/siRNA release, enhanced gene silencing, and reduced tumor microenvironment immunosuppression by ECM hydrogel. The vaccine, composed of LNP/siPD-L1, antigens, and TLR-3 agonists, potently activates and matures DCs, promoting rapid T cell activation and tumor recognition/killing for therapeutic effects against tumors.

### 3.4 ECM hydrogel-based vaccine drives immune responses in lymph nodes

Next, we investigated whether the regulatory effect of Gel + Vaccine also had a corresponding immune-enhancing effect in tumor lymph nodes *in vivo* (Fig. 4a). We examined immune effects in LNs, including CD11c, MHC-I, CD86, CD80, MHC-II, and PD-L1 expression, as well as IL-6 and IL-2 secretion. CD80 (also known as B7–1 or BB1) serves as a critical co-stimulatory molecule for T lymphocyte activation, synergizing with CD86. The interaction of CD80/CD86 with CD28 enhances T lymphocyte activation and clonal expansion. During antigen uptake, DCs will feedback upregulate PD-L1 to suppress T lymphocyte activity. However, encapsulating LNP/siRNA in ECM hydrogel can effectively counteract this immunosuppression and significantly downregulate PD-L1 on DCs. The results show that compared with the normal saline group and the LNP/siRNA group, the gel + LNP group decreased by 25.5% and 10.5% respectively (Fig. 4d, e), highlighting the advantage of the hydrogel-based formulation in the sustained release of LNP/siPD-L1.

**Fig. 4:**
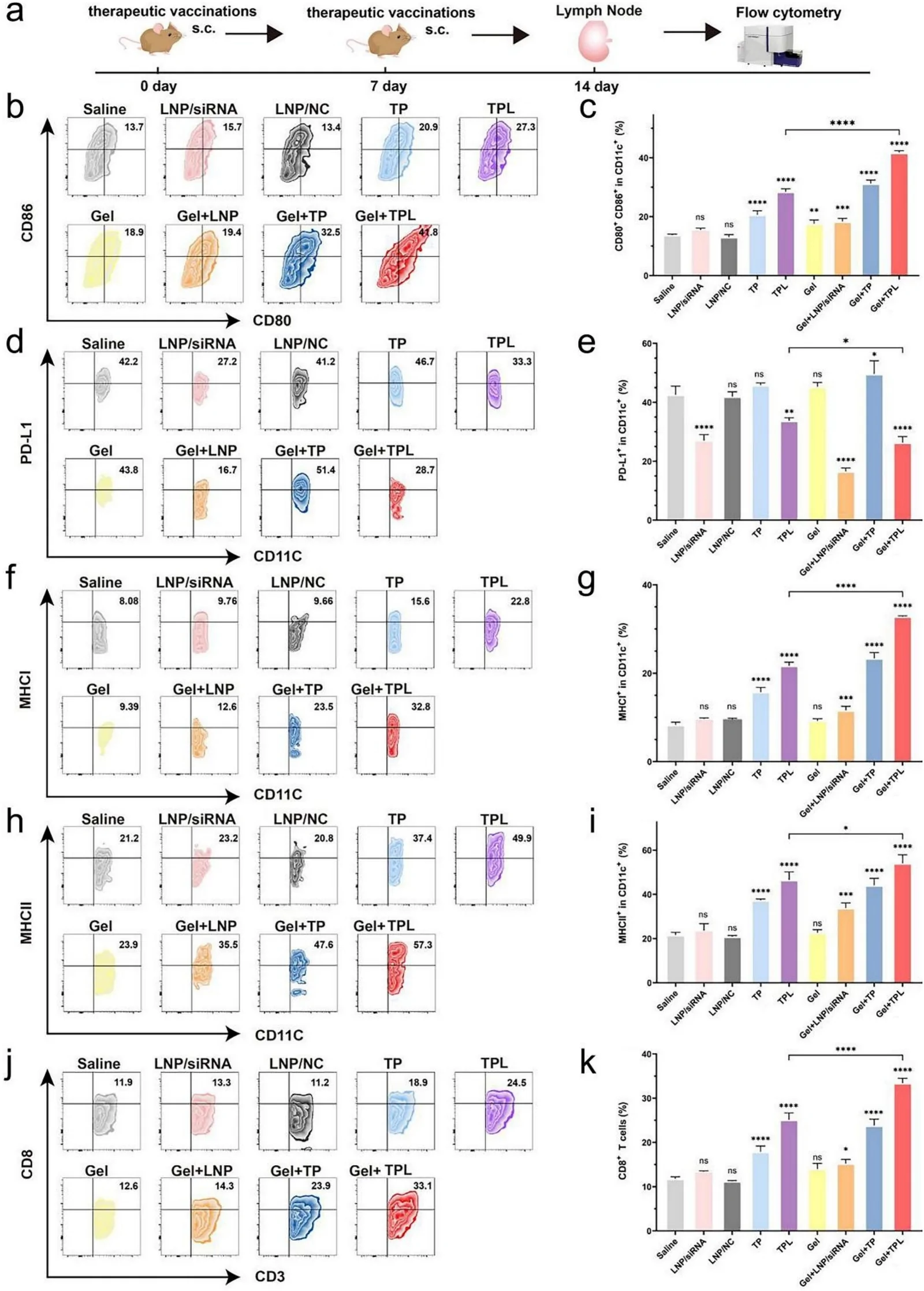
DC Activation Capacity of Treatment Groups in Lymph Nodes. **(a)** Schematic of *in vivo* immunization by various treatment groups in lymph nodes. Representative flow cytometry profiles and corresponding quantitative analysis of **(b-c)** CD80/CD86, **(d-e)** PD-L1 and **(f-g)** MHC-I, and **(h-i)** MHC-II expression on DCs (*n* = 3). **(j-k)** Flow cytometry profiles and) corresponding quantitative evaluation of CD8+ T cell frequency among CD3+ T cells (*n* = 3). Data are mean ± s.d. Statistical significance: one-way ANOVA with Tukey's test. *p < 0.05, **p < 0.01, ***p < 0.001, ****p < 0.0001; ns: no significant difference.

DC antigen presentation was assessed using MHC-I and MHC-II, showing that hydrogel as a vaccine carrier enhances DC immune responses through sustained TCL and poly(I:C) release. As shown in Fig. 4f-i, Gel+TPL increased MHC-I and MHC-II expression by 24.8% and 36.0%, respectively, compared to Saline. Additionally, we analyzed CD8^+^ T cell frequency among CD3^+^ T cells. As shown in Fig. 4j,k, Gel+TPL achieved the highest frequency at 33.1%, outperforming other groups.

These results demonstrate that ECM hydrogel-based cancer vaccines are a superior option for combined immunotherapy. In comparison to both the single-component group and the non-gel formulation group, their therapeutic importance is evident due to enhanced DC activation, CD8^+^ T cell responses in LNs, and TIL expansion when used as a delivery vehicle. These results also provide a basis for further *in vivo* pharmacodynamic studies.

### 3.5 ECM hydrogel-based vaccine triggered effective anti-tumor immune responses

Finally, to assess the therapeutic effectiveness of hydrogel-formulated vaccines, a gastric cancer model was established in 6-week-old male 615 mice. Mice received subcutaneous inoculation of 5 × 10^5^ MFC tumor cells(Zhong et al., 2023), when the tumor volume reaches 100 mm^3^, followed by two vaccinations administered 6 days apart starting on day 8 (Fig. 5a). All mice were monitored throughout the following 20-day experimental period.

**Fig. 5:**
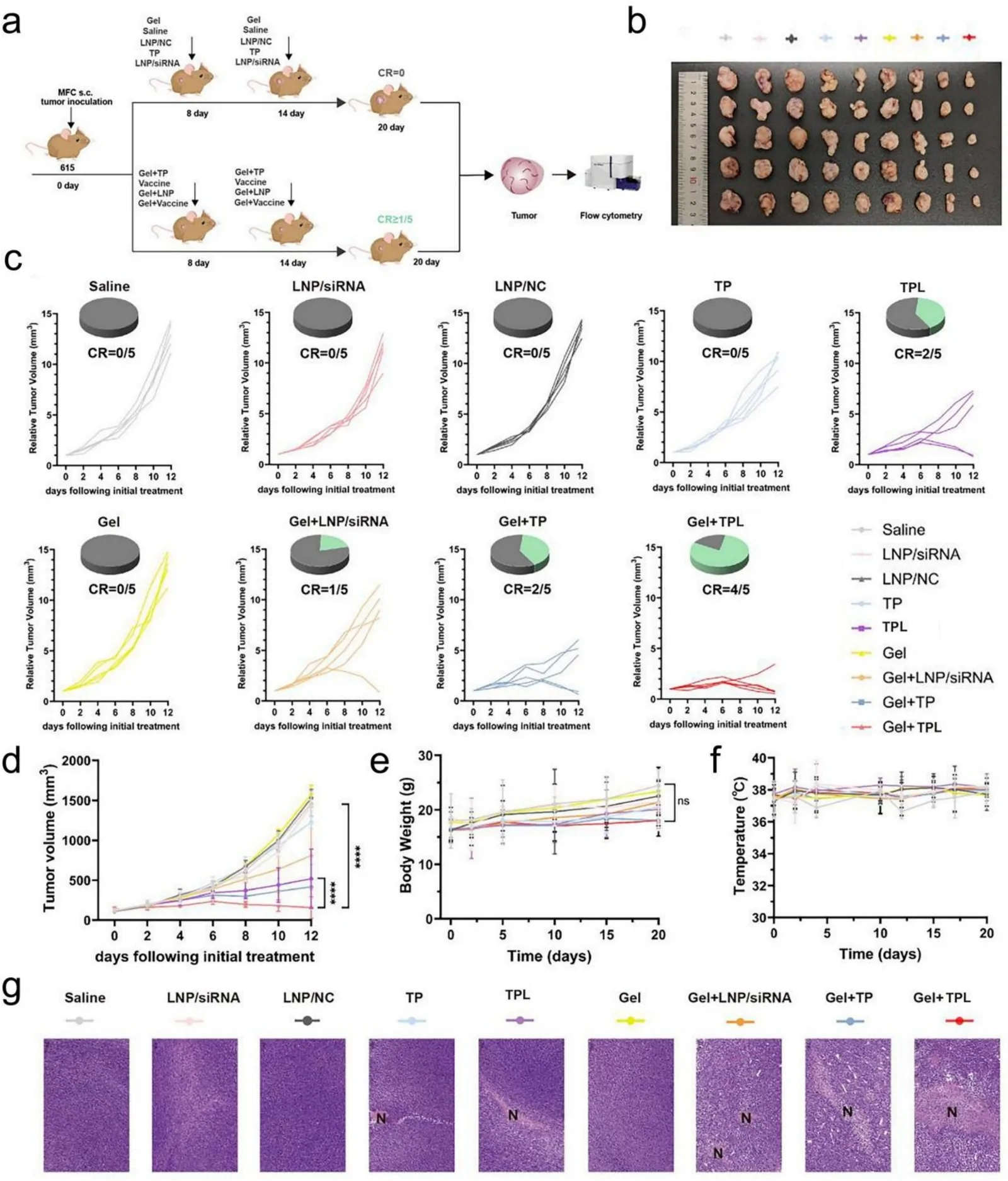
The Antitumor Effect of Hydrogel Vaccine in Mice. **(a)** Schematic of the experimental design and Complete Response (CR) rates (*n* = 5). **(b)** Representative photographs of tumors collected 20 days after tumor inoculation. **(c)** Relative tumor volume and CR rates (*n* = 5). **(d)** Body weight changes over time (*n* = 5). **(e)** Tumor growth curves from the start of treatment (*n* = 5). **(f)** Temperature fluctuations in mice across all treatment groups (*n* = 5). **(g)** HE staining of tumor tissues. The letter “N” indicates necrotic areas.Data are presented as mean ± s.d. Statistical significance was determined using one-way ANOVA with Tukey's test. *p < 0.05, **p < 0.01, ***p < 0.001, ****p < 0.0001; ns: no significant difference.

Compared with other therapeutic groups, the Gel+TPL combination therapy demonstrated superior tumor ablation efficacy. As shown in Fig. 5b-d, Saline, LNP/siRNA, LNP/NC, and Gel groups all exhibited progressive tumor proliferation without any complete responses. In contrast, the Gel+LNP/siRNA group showed tumor growth inhibition, with one complete response observed among treated animals. The TPL, Gel+TP, and Gel+TPL groups effectively delayed tumor growth, achieving 2, 2, and 4 complete responses, respectively. The results demonstrated that the vaccine combination of LNP/siPD-L1, TCL, and poly(I:C) was an effective strategy for treating tumors compared with free components. More importantly, the Gel+TPL group exhibited the strongest tumor suppression ability, indicating that the hydrogel-based formulation was superior to the free formulation. Additionally, no significant difference in body weight change was observed during the 22-day experiment (*p* = 0.053) (Fig. 5e).

No treatment group exhibited significant fluctuations in body temperature, indicating the absence of systemic immune-inflammatory responses (Fig. 5f). Moreover, the hearts, kidneys, lungs, livers, and spleens of mice across different treatment groups were subjected to H&E staining for histopathological evaluation. As illustrated in **Fig. S6**, comparative analysis with the saline control group revealed no significant pathological alterations in the organ morphology of mice treated with the drug. These findings suggest that the dECM hydrogel-based tumor vaccine exhibits favorable biocompatibility and biosafety.

HE staining of tumor sections revealed extensive apoptosis/necrosis in the Gel+TPL group, as evidenced by nuclear collapse and cytoplasmic rupture (Fig. 5g). The ‘N' denotes necrotic areas within the tumor tissue. In the Gel+TPL group, tumor sections exhibited prominent eosinophilic regions (pink cytoplasmic staining) accompanied by nuclear pyknosis/fragmentation due to loss of hematoxylin staining. These pathological alterations align with the morphological criteria for complete tumor necrosis outlined in the WHO guidelines for tumor response evaluation(Wolchok et al., 2009), indicating nearly complete tumor cell lysis induced by this treatment regimen.

### 3.6 ECM hydrogel-based vaccine enhances anti-tumor immune activity

Furthermore, to further analyze the therapeutic mechanism, we analyzed T cell infiltration in tumor tissues. The results demonstrated that the Gel+TPL formulation efficiently recruited tumor-infiltrating T cells. Specifically, the proportion of CD4+ T cells within the Gel+TPL group was significantly higher compared to other groups: 3.88-, 2.74-, 3.56-, 1.56-, 1.32-, 2.69-, 1.75-, and 1.30-fold higher than those in the Saline, LNP/siRNA, LNP/NC, TP, Vaccine, Gel, Gel+LNP/siRNA, and Gel+TP groups, respectively (Fig. 6a,b). Furthermore, we assessed infiltration of CD8^+^ T lymphocytes. The percentage of CD8^+^ T cells in the Gel+TPL group was 3.64-, 2.60-, 3.45-, 1.98-, 1.46-, 2.88-, 2.12-, and 1.37-fold higher than those in the Saline, LNP/siRNA, LNP/NC, TP, Vaccine, Gel, Gel+LNP/siRNA, and Gel+TP groups, respectively (Fig. 6c,d).

**Fig. 6:**
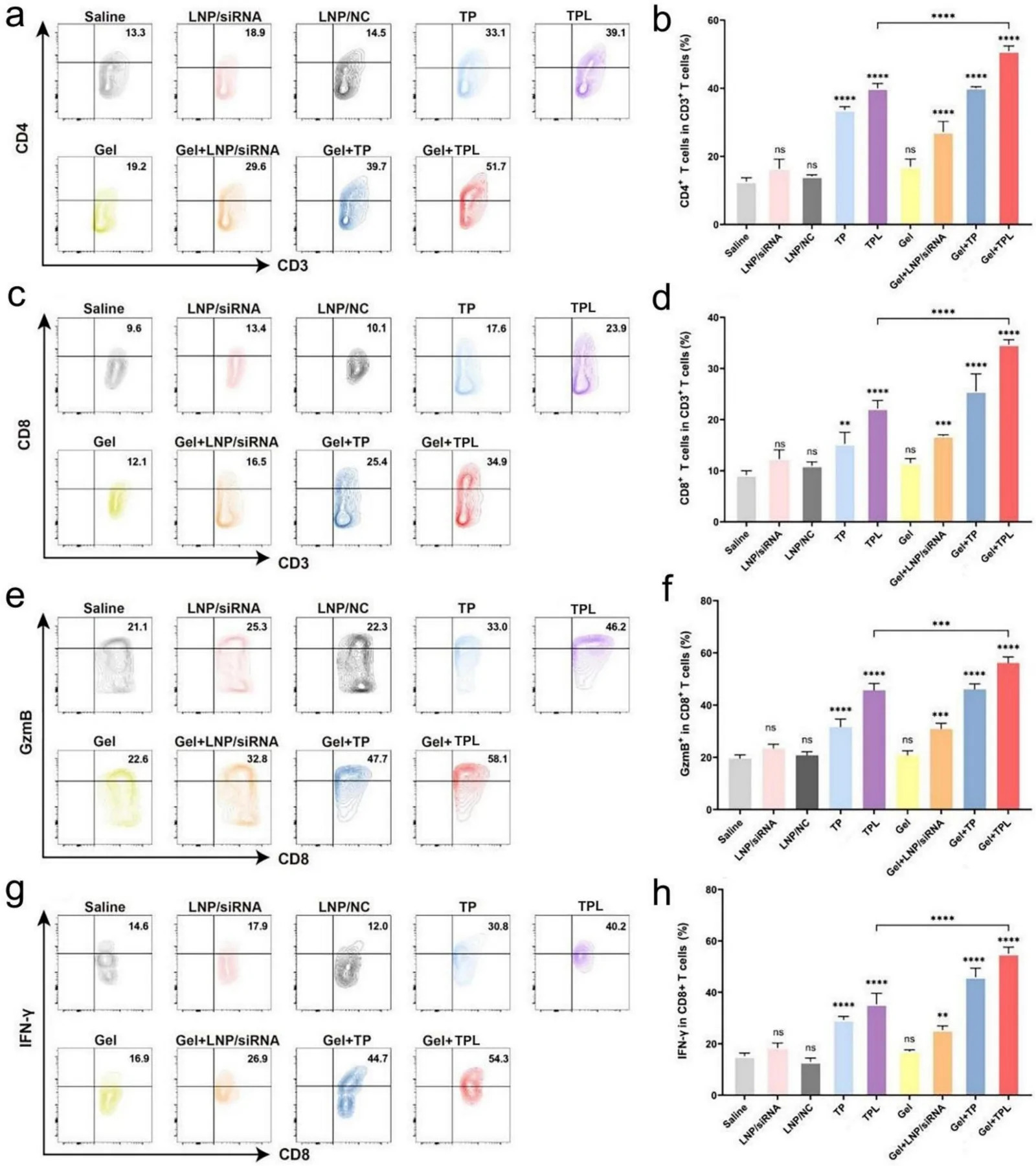
Hydrogel vaccine induces robust Tumor-infiltrating T lymphocyte immunity. **(a)** FCM plots and **(b)** percentages of CD4^+^ T cells among CD3^+^ T cells in tumors (*n* = 3). **(c)** FCM and **(d)** percentages of CD8^+^ T cells among CD3^+^ T cells (*n* = 3). **(e)** FCM and **(f)** percentages of GzmB^+^ CD8^+^ T cells (*n* = 3). **(g)** FCM and **(h)** percentages of IFN-γ^+^ CD8^+^ T cells (*n* = 3). Data are mean ± s.d. Statistical significance: one-way ANOVA with Tukey's test. *p < 0.05, **p < 0.01, ***p < 0.001, ****p < 0.0001; ns: no significant difference.

The secretion of inflammatory cytokines, including IFN-γ and GzmB by CD8^+^T cells plays a crucial role in their anti-tumor efforts. As shown in Fig. 6e-h, the populations of IFN-γ^+^ and GzmB^+^ CD8^+^ T cells in tumor tissues from mice treated with Gel+TPL (GzmB^+^: 58.1%, IFN-γ^+^: 54.3%) were markedly increased compared to those treated with Saline (GzmB^+^: 21.1%, IFN-γ^+^: 14.6%), LNP/siRNA (GzmB^+^: 25.3%, IFN-γ^+^: 17.9%), LNP/NC (GzmB^+^: 22.3%, IFN-γ^+^: 12.0%), TP (GzmB^+^: 33.0%, IFN-γ^+^: 30.8%), Vaccine (GzmB^+^: 46.2%, IFN-γ^+^: 40.2%), Gel (GzmB^+^: 22.6%, IFN-γ^+^: 16.9%), Gel+LNP/siRNA (GzmB^+^: 32.8%, IFN-γ^+^: 26.9%), and Gel+TP (GzmB^+^: 47.7%, IFN-γ^+^: 44.7%). These findings indicate more effective *in vivo* activation of CD8^+^ T cells by Gel+TPL.

Importantly, CD8^+^ T cells activated by vaccines could differentiate into memory T cell subsets critical for long-term immunity. As shown in **Fig. S5**, Gel+TPL treatment also enhanced the populations of central memory T cells (T_CM_) and effector memory T cells (T_EM_) in tumor tissues. Compared to other groups, the Gel+TPL group exhibited a 1.80-, 2.15-, 2.17-, 1.37-, 1.28-, 2.31-, 1.45-, and 1.18-fold increase in T_CM_ frequency and a 3.58-, 3.02-, 3.05-, 2.14-, 1.73-, 2.68-, 2.72-, and 1.42-fold increase in T_EM_ frequency, respectively. Collectively, these results demonstrate that Gel+TPL not only enhances acute CD8^+^ T cell activation but also alters the number of cells that display memory-related phenotypes.

## 4 Discussion

The immune system of the human body is extremely complex and has a powerful ability to resist the invasion of foreign pathogens, eliminate abnormal cells and maintain the homeostasis of the internal environment. In recent years, many breakthroughs have been made in the design and development of therapeutic vaccines for tumors, greatly promoting the clinical application of vaccines(Huang et al., 2022; Jou et al., 2021; Morse et al., 2021).

In the design of tumor vaccines, although the selection of antigens and adjuvants remains a core factor, more and more studies have shown that the rational design of delivery systems and the choice of vaccination routes are equally important in determining the overall efficacy of vaccines. The integrated design of “delivery-activation-immunostimulation” may become an important potential path for therapeutic vaccines for tumors to truly achieve clinical application(McNeel, 2018).

Intelligent spatiotemporal control of the *in vivo* pathways and release processes of vaccines, releasing antigens and adjuvants at the appropriate location and within the suitable time window, can maximize the activation of anti-tumor immune responses. In terms of spatial control, targeting the paracortical area of lymph nodes and promoting the endocytosis of dendritic cells can be achieved through the rational design of carriers. Regarding temporal control, moderately extending the antigen release time (*e.g.*, several days to several weeks) or adopting a “pulse + maintenance” dual-phase release mode is expected to more effectively activate the immune system, alter the number of cells exhibiting memory-related phenotypes, and reduce the risk of tumor recurrence and metastasis. Therefore, an integrated delivery system combining spatial and temporal control may simultaneously achieve targeted delivery, sustained release, and immune activation, exerting a synergistic effect, and represents a new vaccine design strategy(Gong et al., 2024; Jiang et al., 2024; Senti et al., 2024).

Based on these insights, we developed an injectable extracellular matrix hydrogel vaccine platform that integrates three components in time and space: TCL, poly(I:C), and PD-L1 siRNA encapsulated in LNPs. This hydrogel vaccine can effectively deliver antigens while relieving immune suppression in DCs, thereby achieving more effective activation of recruited DCs, amplifying tumor-specific CD8+ T cell priming, and ultimately driving a strong anti-tumor immune response(Holder et al., 2024).

Current research still has some limitations. Firstly, although porcine-derived biomaterials have been widely used in tissue engineering, such as basic scaffolds (bone/skin), and even explored as sources for complex organ (kidney/liver) transplantation(Barajaa et al., 2024; Nguyen et al., 2024). There are few reports on their use as vaccine scaffolds. Further evaluation of their biocompatibility in non-human primate models is needed. Secondly, although tumor cell lysates are rich in antigenic epitopes, there are still issues such as epitope drift and reduced immunogenicity. Some emerging methods, such as protein degradation using proteolysis-targeting chimeras (PROTAC), AI-guided neoantigen identification, and multi-omics analysis, may help understand the mechanisms and seek solutions to these problems(Bender and Cortés-Ciriano, 2021; Zeng et al., 2025). Thirdly, although PD-L1 plays a core role in DC-mediated immunosuppression, the molecular interactions between tumor-expressed immunosuppressive molecules and DCs are extremely complex, thus leading to insufficient efficacy. Some new strategies, such as bispecific antibodies targeting both transforming growth factor-β (TGF-β) and PD-L1, reversing the metabolic reprogramming of DCs and restoring oxidative phosphorylation (OXPHOS), may further enhance the efficacy when used on this platform(Gulley et al., 2022). In addition, comparative studies of various administration routes should be conducted. Vaccines can be administered through different routes, such as intramuscular, subcutaneous, intradermal, intravenous, intratumoral, and mucosal administration. The administration route can affect the efficacy and safety of vaccines. Although subcutaneous injection, which was used in this study, has shown enhanced targeting of lymph nodes and increased neoantigen-specific T-cell responses, mucosal administration methods such as nebulization, intranasal, or sublingual administration may also have great potential due to the natural mucosal adhesiveness of hydrogels(Eshaghi et al., 2024; Tscherne and Krammer, 2025). Moreover, the enhanced immune response observed may result from a potential synergistic effect between multi-component vaccines and the ECM microenvironment, rather than a single mechanism. However, the current study design has not quantitatively disentangled the individual contributions of lipid nanoparticle retention, ECM-specific biochemical signaling, matrix mechanics, and degradation products to therapeutic efficacy. This limitation could be addressed in future studies through experimental approaches such as release-matched controls, ECM composition controls, and systematic analysis of local innate and adaptive immune responses, along with further investigation into the specific mechanisms underlying carrier retention and changes in biochemical signaling.

## 5 Conclusions

Overall, this collection platform has initially demonstrated great potential as a personalized cancer immunotherapy. The enhanced antitumor efficacy observed with the porcine lung decellularized ECM hydrogel-based vaccine is likely attributable to the integrated effects of the matrix and its immunologically active cargo. The tumor cell lysate provides a broad antigenic source, whereas poly(I:C) can promote innate immune activation and support dendritic-cell maturation and cross-presentation. The siRNA component may further regulate the targeted immunosuppressive or tumor-associated pathway. Within this formulation, the ECM hydrogel may serve not only as a delivery matrix but also as an immunomodulatory microenvironment. By retaining vaccine components locally, the matrix may prolong antigen and adjuvant exposure and improve the local availability of siRNA. Meanwhile, ECM-derived proteins, glycosaminoglycans, and matrix-associated factors may regulate immune-cell adhesion, migration, antigen uptake, and activation. Matrix remodeling may additionally release bioactive ECM fragments and modify local inflammatory signaling. The three-dimensional matrix may also affect the spatial organization and interaction of antigen-presenting cells and lymphocytes.

To achieve clinical translation, a large amount of research still needs to be carried out, and there are uncertainties. Future research will focus on optimizing the physical and chemical properties of the hydrogel system (such as pore structure and degradation kinetics) and their impact on immune activation, exploring the synergistic effects between the vaccine and traditional treatment methods (such as radiotherapy and chemotherapy), evaluating the immunogenicity and protective effects in large animal models (including pigs and non-human primates), and conducting a comprehensive assessment of safety and long-term immune memory effects, *etc.*

## CRediT authorship contribution statement

**Jie Wu:** Writing – original draft, Investigation, Formal analysis, Data curation. **Xinxin Bu:** Methodology, Investigation, Formal analysis, Data curation. **Zi Liu:** Methodology, Investigation, Formal analysis, Data curation. **Yupeng Zhou:** Methodology, Investigation, Formal analysis, Data curation. **Lingzhi Zhang:** Methodology, Investigation, Data curation. **Zixuan Wang:** Methodology, Investigation, Formal analysis, Data curation. **Wenquan Cheng:** Methodology, Investigation, Formal analysis. **Yanxia Yu:** Writing – review & editing, Writing – original draft, Supervision, Methodology, Conceptualization. **Huae Xu:** Validation, Supervision, Methodology, Investigation, Conceptualization. **Qun Gu:** Writing – review & editing, Methodology, Investigation, Formal analysis, Conceptualization.

## Ethics declaration

This study was conducted in accordance with the ARRIVE (Animal Research: Reporting of *In Vivo* Experiments) guidelines. This study was approved by the Institutional Animal Care and Use Committee of NMU. (Approval No. IACUC-2507020)

## Declaration of competing interest

The authors declare that they have no known competing financial interests or personal relationships that could have appeared to influence the work reported in this paper.

## Data Availability

Data will be made available on request.
