# Supplementary material for: Biomimetic nano-in-gel vaccine platform with spatiotemporal co-delivery capacity for DC maturation against immunosuppressive tumors
Source: Int J Pharm X. 2026 Sep 16;12:100662. doi: 10.1016/j.ijpx.2026.100662 (PMC13629215; doi:10.1016/j.ijpx.2026.100662)
Supplement: Supplementary file 1 [file mmc1.docx]

**Biomimetic Nano-in-Gel Vaccine Platform with Spatiotemporal Co-Delivery Capacity for DC Maturation against Immunosuppressive Tumors**

Jie Wu^1#^, Xinxin Bu^1#^, Zi Liu^2,3#^, Yupeng Zhou^1^, Lingzhi Zhang^1^, Zixuan Wang^1^, Wenquan Cheng^1^, Yanxia Yu^2,3*^, Huae Xu^1*^, Qun Gu^4,5*^

1 Department of Pharmaceutics, National Vaccine Innovation Platform, School of Pharmacy, Nanjing Medical University, Nanjing 211166, China.

2 Department of Pharmacy, The Affiliated Suzhou Hospital of Nanjing Medical University, Suzhou Municipal Hospital, Suzhou, People’s Republic of China

3 Gusu School, Nanjing Medical University, Suzhou, People’s Republic of China

4 Department of Cardiovascular Surgery, People’s Hospital of Kizilesu Kirgiz Autonomous Prefecture, Artux City, People’s Republic of China

5 Department of Cardiovascular Surgery, The First Affiliated Hospital with Nanjing Medical University, Nanjing 210029, China

Corresponding author: [yuyanxia@njmu.edu.cn](mailto:yuyanxia@njmu.edu.cn) (Y Yu); [xuhuae@njmu.edu.cn (H Xu);](mailto:xuhuae@njmu.edu.cn;) [guqundr@njmu.edu.cn](mailto:guqundr@njmu.edu.cn) (Q Gu)

**Supplementary data**

**Figure S1** The influence of different concentrations, pH and ionic strength on ECM hydrogel formation. From left to right, the concentration of ECM solution were 6mg/ml, 8mg/ml, 10mg/ml, 12mg/ml, 15mg/ml and 20mg/ml. **a)** ECM hydrogel formation with different concentrations, pH=1.0 and physiological salt concentration (10X PBS). **b)** ECM hydrogel formation with different concentrations, pH=7.0 and physiological salt concentration (10X PBS). **c)** ECM hydrogel formation with different concentrations, pH=7.4 and physiological salt concentration (10X PBS). **d)** ECM hydrogel formation with different concentrations, pH=8.0 and physiological salt concentration (10X PBS).

These results indicated that the ECM solution with pH=1.0, 7.0 and 8.0 could not form a hydrogel, and even the ECM solution adjusted to pH=7.4 without 10X PBS was failed to form hydrogel. 12 mg/mL ECM solution could form a gel in 5 min at 37°C with extremely poor mechanical strength, When the sample bottle was turned upside down, the hydrogel fell out slowly. Only when the ECM solution were 15mg/ml and 20mg/ml, equipped with pH=7.4 and 10X PBS, the ECM hydrogel could formed successfully in 3 min at 37°C with good mechanical strength. Eventually, we chose 15 mg/mL ECM solution for the following experiments to save materials.

**Figure S2** The influence of temperature on ECM hydrogel formation.

The ECM solutions at 4℃ and 45℃ were liquid, while the ECM solution at 37℃ could formed a hydrogel. All these results confirmed the temperature sensitivity of the ECM hydrogel. It could not form a hydrogel at 45°C, which may be caused by protein denaturation at higher temperatures.

**Figure S3** Viability of DCs treated with different concentrations of ECM hydrogel (n = 5).

**Figure S4** Biosafety of ECM hydrogel in C57BL/6J mice. **a**) Liver indicators included Alanine Aminotransferase(ALT), Aspartate Aminotransferase(AST), Total Bilirubin（T-BIL) (n=3). Kidney indicators included Creatinine(CR) and Blood Urea Nitrogen(BUN) (n=3). **b**) The indicators of blood routine included White Blood Cell(WBC), Red Blood Cell(RBC), neutrophilicgranulocyte percentage(Gran%), Lymphocyte percentage(Lymph%) and Mononuclear cell percentage(Mon%). **c**) Inflammatory response of subcutaneous tissue in different volume treated mice for 9 days.

The liver and kidney indicators of the mice in each group were normal and no significant statistical difference. This proved that the ECM hydrogel did not damage the liver and kidney function of mice. No inflammatory response was observed in the subcutaneous tissue when the injection volume of the hydrogel was 100 μL. When the injection volume of the hydrogel reached 200 μL, the inflammatory response of the subcutaneous tissue was only limited to three days, and then was relieved. Then, when the injection volume of hydrogel reached 300μl, the inflammatory response of subcutaneous tissue was relatively strong. Thus, we should control the injection volume hydrogel below 300μl.

**Figure S5 a)** Representative flow cytometry plots and **b)** bar chart of central memory T cells (T_CM_) and effector-memory T cells (T_EM_) in CD8^+^ T cells isolated from tumors (n = 3).

**Figure S6** Histopathological staining sections of organs in mice treated with each treatment groups.
